# “Motherhood is a work in progress”: a qualitative longitudinal study of maternal subjectivity and well-being of women in the Bukhali trial in Soweto, South Africa

**DOI:** 10.3389/fgwh.2026.1880613

**Published:** 2026-08-31

**Authors:** Neusa F. Torres, Daniella Watson, Lerato Mohlomi, Tsakani Hlungwani, Caitlin V. Gardiner, Noreth Muller-Kluits, Shane A. Norris, Catherine E. Draper, Michelle Pentecost

**Affiliations:** 1 SAMRC/Wits Ageing African Adult Research Unit, Department of Paediatrics, School of Clinical Medicine, Faculty of Health Sciences, University of the Witwatersrand, Johannesburg, South Africa; 2 Department of Global Health and Social Medicine, King's College London, London, United Kingdom; 3 Instituto Superior de Ciências de Saúde (ISCISA), Maputo, Moçambique; 4 School of Psychology, University of Surrey, Surrey, United Kingdom; 5 School of Human Development and Health, University of Southampton, Southampton, United Kingdom

**Keywords:** identity, matrescence, motherhood, qualitative longitudinal research, Soweto, well-being, women

## Abstract

**Background:**

Motherhood is a crucial yet understudied aspect of maternal health, especially in sub-Saharan Africa, where inequalities and focus on clinical outcomes impact care. The concept of maternal subjectivities and matrescence, has limited exploration in South African public health. This article uses qualitative longitudinal data from the Bukhali trial to examine the social dynamics that shape maternal identities among young women.

**Methods:**

Sixty women, mostly single, aged 22–34, participated in multiple interviews from 2023 to 2025. Using Neale's (2021) approach, we created case profiles and selected four for detailed analysis. Data were analysed with inductive coding and longitudinal strategies.

**Results:**

Women's experiences of motherhood revolve around four interconnected themes. The first involves the emotional rollercoaster of matrescence and motherhood, described as intense and unpredictable, with love, exhaustion, pride, and self-doubt happening simultaneously. The second theme highlights financial struggles and economic instability, common among those facing unemployment, informal work, or limited social grants. The third theme emphasises the necessity of sacrifice and re-evaluating personal ambitions, an everyday part of motherhood rather than an exception, requiring balance between personal goals and children's immediate needs. The fourth stresses the crucial role of social support: its presence is vital, while its absence can lead to feelings of isolation, postnatal depression, and parenting difficulties for single mothers.

**Conclusion:**

Motherhood in Soweto is complex and influenced by structural constraints and change. The caregiver-earner dual burden reflects post-apartheid gendered labour, while kinship support varies, challenging assumptions of female solidarity. Participants valued health education related to trials but reported no support for motherhood transition. The study recommends maternal-centered programs that address women’s psychosocial needs, personal experiences, and combine emotional and peer support with medical care.

## 1 Introduction

**“**I'm still trying to figure out who I am, the one who is a mother, because there is a shift. What is it that I like about being me now? What does it mean to be a mother? I'm trying to be a better version of myself for my son. It is a lot; it is a work in progress” (Phumi, wave 3).

Motherhood brings significant changes to identity, relationships, and self-perception in ways that are often unimaginable and profoundly influence one's sense of self, personal aspirations and life goals (1). The experience of such changes is referred to as ‘maternal subjectivity’: the distinctive form and perception of selfhood that emerges through becoming and being a mother, includes complex and multifaceted experiences, emotions, and perspectives that are related to one of the identity, agency, and embodiment during pregnancy, childbirth, and motherhood (2–5). The concept recognises that motherhood is far from being a one-size-fits-all experience, and that each mother possesses her own set of emotions, thoughts and identity in the journey of parenthood, which is, importantly, dynamic over time (5–12). A key art of this journey is that of matrescence, the developmental change a woman goes through in becoming a mother, which is a time when women are especially vulnerable (13). Yet, research in maternal and child health largely frames mothers as standardised objects of intervention at cross-sections in time, rather than as women whose temporally dynamic and shifting subjective experiences powerfully influence how successful any intervention might be.

In South Africa, the experience and enactment of motherhood have always taken distinct forms because of the effects of changing social and political realities wrought by colonialism, apartheid and its aftermath. For example, housing and labour migration policies over the twentieth century produced social formations that had a major impact on the structure and functioning of ‘the family’ and child-rearing norms (14–16). Now, post-apartheid, unemployment in South Africa is extremely high, often requiring women to work outside the home and therefore not be available to care for their children full-time (7, 17). Additionally, and partly related to these structural injustices and obstacles, mental health difficulties during pregnancy and postpartum are common. It is also well documented that the transition to motherhood can provoke anxiety, depression, or existential reflection, profoundly affect relationships, and induce feelings of isolation and inadequacy (18, 19). In light of these factors, studies show that about 20%–35% of South African women experience significant symptoms of depression or distress in the peri- and postnatal periods (20–22). When left untreated, such distress is linked to poorer mother–infant bonding and worse child outcomes, highlighting the importance of early recognition and support, which is often lacking in healthcare systems. Furthermore, social support can protect the physical and emotional well-being of those exposed to stress through a buffering effect, thereby reducing psychological distress, depression and anxiety (23, 24). Notably, even the mere *perception* of support improves health and well-being by reducing stress levels (25, 26). The social network, or what Pillay calls ‘kinship capital’, plays a key role in support for mothers, especially in low-income urban spaces (17). Mental health and the factors surrounding it are integral to the experience of motherhood, and vice versa; thus, the failure to engage with maternal subjectivity as central to women's health can negatively affect a women's self-esteem and well-being.

While there is a robust and growing international qualitative literature on maternal subjectivities (predominantly focused on Global North contexts) (27–29), there is a considerably lower amount that draws on qualitative *longitudinal* research (QLR), a significant gap given the highly dynamic nature of maternal subjectivities over time. There is a similar pattern in the South African literature: while there is a growing corpus of social science research that attends to the diversity of maternal subjectivity (30–36). Research drawing on qualitative longitudinal data is very limited and tends to make the maternal subjectivity contingent on other topics (for example, Moses and Tomlinson, 2012, and their focus on HIV disclosure) (37). In this study, we thus make the important contribution of using QLR to investigate the concept of maternal subjectivities itself in the South African context of Soweto. While significant empirical research from Soweto cohorts has expanded the literature on a range of topics, including adolescent pregnancy risk factors, perinatal social support, pregnancy termination decision-making, and postpartum mental health among Soweto mothers (38–41). There has been little longitudinal work on how young women in Soweto experience motherhood as a lived identity.

In addition, in this research on maternal subjectivities, we contribute to the literature by shifting the frame from a focus on women as points of contact for intervention for maternal and child health to a focus on *African* women as *subjects* in their own right. Our impetus for such work is twofold: (1) to conduct research that represents the heterogeneity of social realities present and (2) to conduct research that prioritises personal subjectivities to, in the words of Elaine Salo, “recuperate Africans’ marginalised subjectivities” and “complicate our representations of African diversity” (36). In addition to a turn away from the image of marginalised Africa, Salo called for close attention to our positionality as researchers producing situated knowledge and to the nuanced temporalities of African life that do not conform to universal linearity (36). Salo argued specifically for anthropology and feminism to avoid the analytical error of “failure to examine the finer multiple, layered temporal and spatial contexts that shape or are informed by local cultural practices and gendered social relations” (36). We thus draw on sociological and anthropological studies of motherhood in South Africa that laid the foundation for understanding motherhood as discourse, practice, and social identity, and that understand the transition to motherhood in both life-course and intergenerational frames (42, 43).

In this research, we aim to examine the relational and social dynamics that shape maternal subjectivities in Soweto, which need to be accounted for in health policy and programme planning. We do this by drawing on qualitative longitudinal data with women participating in the South African cohort of the Healthy Life Trajectories Initiative (HeLTI). HeLTI is an international consortium of life course trials with cohorts in Canada, China, India and South Africa (44)). HeLTI is grounded in the science of the Developmental Origins of Health and Disease (DOHaD), which holds that early-life interventions are the most effective for lifelong health outcomes (45). The trial is thus testing a four-phase complex intervention, primarily with young women, across the continuum of preconception, pregnancy, infancy, and early childhood periods to assess its effects on childhood obesity (primary outcome) and other maternal and child health and developmental outcomes (secondary outcomes) (44). The complex integrated intervention includes micronutrient supplementation and specific behaviour change interventions, such as repeated counselling sessions. The control groups for each cohort receive an enhanced ’standard of care’ package, including usual state-mandated care and general lifestyle advice, all adapted to context (46).

The HeLTI-South Africa cohort, also known as “Bukhali” (a Zulu word for smart/powerful), has recruited a cohort of 6,360 women aged 18–28 in Soweto, Johannesburg. Bukhali has aimed to be pragmatic and adapt to context, having to navigate substantial and diverse participant priorities and basic needs, low levels of health literacy, and difficulties in participant contactability and migration (46, 47). Notably, Bukhali is the first intervention trial in Africa to include a preconception health component; it is thus well-suited to longitudinally examine women's transition to motherhood from before to after motherhood. Additionally, a core aim of Bukhali is to inform public health policy in South Africa (46).

Considering this and the nuanced picture of maternal subjectivity we present in this context, we argue for a focus on maternal subjectivity rather than maternal risk in framing public health interventions derived from cohort studies such as HeLTI. We especially argue for a strong public health focus on matrescence—the period of transition to motherhood—as a time of particular vulnerability when attention to maternal subjectivities is required.

## 2 Methods

### 2.1 Study setting

Soweto is a low-income, densely populated urban settlement in Gauteng province, South Africa. Its name derives from the first two letters of each word in the phrase ’South-Western Townships’, So-We-To. Soweto was formed as part of apartheid's segregation policy, when inhabitants classified as Black were obliged to move away from, but still work in the economic centre of the city of (48). Soweto became the largest “township” in the country and is characterised by ethnic, linguistic, and cultural diversity. Many residents speak multiple languages, including IsiZulu, IsiXhosa, Setswana, Sesotho, Xitsonga, English, and Afrikaans. Economically, levels of wealth and income vary in Soweto. The majority live in lower-income neighbourhoods, where corrugated iron dwellings, shared toilets, and unpaved roads are common (49).

Women in Soweto face numerous challenges, including crime, unemployment, poverty, malnutrition, and food insecurity. The health profile of Soweto is characterised by multiple comorbidities, including high rates of HIV/AIDS, type 2 diabetes, obesity, and hypertension. Unintended pregnancies account for 56.5% among women aged 20–24 years. Women experience stress, depression, poor sleep quality, and anxiety, with these effects amplified in the perinatal period (39, 50–56).

### 2.2 Study design

This article draws on data from a qualitative longitudinal study conducted as part of the Trajectories sub-study of the Bukhali trial in Soweto, South Africa. The Trajectories study follows women over time to understand how their lives unfold, including transitions into adulthood, pregnancy, and motherhood, and how social, economic, and relational contexts shape these transitions. A qualitative longitudinal approach was used to capture change and continuity in women's experiences through repeated in-depth interviews. For this article, we focus on data from three waves of interviews conducted between 2023 and 2024, paying particular attention to how women experienced relationships, education, employment, finances, life goals, and parenthood, including becoming and being mothers, and how these experiences shaped their well-being and everyday lives.

### 2.3 Ethics

Ethical approval for this study was granted by the University of the Witwatersrand Human Research Ethics Committee (Medical) (M210157) on 26 February 2021. At the outset, participants received a consent form and an information sheet outlining the study's purpose, process, and potential outcomes. Written consent was obtained during the first interview, with verbal consent recorded for subsequent waves. As with all contact points in the HeLTI trial, participants received a snack and reimbursement for transport costs after each interview.

### 2.4 Sampling and recruitment

For this Trajectories study, participants were recruited from the Bukhali trial, which enrols women aged 18–28 years living in Soweto. Recruitment for Bukhali was conducted through a range of community-based strategies, including social media, door-to-door visits, and community outreach activities (57, 58).

Women in the Trajectories qualitative longitudinal study were recruited telephonically. The qualitative cohort initially comprised 60 women who participated in the first round of interviews (February–May 2023). Of these, 45 took part in the second round (September–October 2023), 32 in the third round (April–May and September–October 2024), and 41 in the fourth round (March–April and August–September 2025). Some attrition across the waves was due to participants changing their contact numbers, relocating, or declining an interview due to new work or childcare commitments. None of the participants who did not attend the follow-up study formally withdrew from the study; rather, they declined an interview for practical reasons but consented to be contacted for future waves. Attrition was thus attributed to structural rather than study-related factors.

### 2.5 Data collection

Data were generated through repeated, in-depth, face-to-face interviews conducted by experienced qualitative researchers (*N*T, DW, LM, TH). Participants’ first interview used a life-history approach, inviting women to discuss their childhoods, education, relationships, work, hopes for the future, and current life circumstances, including their experience of participating in the trial. At the end of the first interview, participants were asked which topics they would most like to discuss in future interviews. Many highlighted motherhood/parenthood, family life, relationships, education, finances, and life plans and goals. These priorities informed the interview guides for subsequent waves, which were developed and refined iteratively.

All interviews took place at the research unit of Chris Hani Baragwanath Academic Hospital, where participants attended the Bukhali trial. Interviews were conducted in participants’ preferred languages, including English, IsiZulu, Setswana, Sesotho, and Xitsonga. Each interview was conducted by two researchers to ensure effective communication and mutual understanding, particularly when multiple languages were used. Interviews lasted between 45 and 90 min, were audio-recorded, transcribed verbatim, and translated into English where necessary. One researcher typically led the interview, while the other served as an observer and interpreter as needed. After each interview, the research team held a short debriefing session and produced reflection and observation notes. The reflection notes captured the interviewer's impressions, emotional responses, and emerging questions, while the observation notes documented the interview setting, participants’ interactions, and non-verbal communication. These materials informed subsequent interviews and were analysed alongside the interview transcripts.

### 2.6 Data analysis

We conducted qualitative longitudinal analysis (QLA), following Neale's approach (2021). A detailed case profile and summative pen portrait were generated for each participant from the interview transcripts, reflection notes, and observation notes. These were shared and discussed in the research group. Pen portraits are concise narrative summaries (one page) that capture key moments from each interview and situate participants’ experiences within their broader life contexts. Case profiles are more detailed documents that include life histories, temporal trajectories, and reflections on past experiences, present circumstances, and anticipated futures. In line with QLR principles of iterative and temporal analysis, pen portraits and case profiles were updated after each wave of data collection to document continuity and change in participants’ lives.

This analytic work took place between interview rounds, allowing the team to reflect on emerging patterns, track developments over time, and refine the focus for subsequent interviews. Working across transcripts, pen portraits, and case profiles enabled engagement with the data at multiple analytic levels and supported a coherent longitudinal interpretation of participants’ trajectories. This process helped us to organise, synthesise, and make sense of the growing longitudinal data set. This approach captures both depth and change over time by working across cases, themes, and temporal sequences. It enabled us to build a rich, layered understanding of participants’ lives and the broader social contexts that shape their experiences.

#### 2.6.1 Coding procedures and theme development

The team conducted both wave-by-wave and longitudinal analyses, enabling thematic mapping and understanding of change over time. NT conducted a thematic analysis of Wave 1 interview transcripts from all 60 participants. These life course histories identified the experience of motherhood or the transition to motherhood as a key theme for this cohort. The team then identified a set of cases to examine longitudinal patterns related to motherhood, maternal subjectivity, and the role of a mother as mentioned by participants. Case inclusion criteria were engagement across at least three interview waves and rich data on motherhood and maternal subjectivity. Sixteen cases were identified for in-depth longitudinal case analysis. Of this set, four cases are used as emblematic cases in this article. Emblematic cases were chosen based on their theoretical relevance (that is, to maternal experiences and subjectivities), their data richness and narrative depth, and their temporal dynamics, which mapped changes in subjectivities (Neale, 2021). Following Neale's (2021) concept of “cases that shine,” the final four cases are selected for their depth and ability to illustrate the heterogeneity of maternal subjectivities across social support, economic situation, relationship status, and emotional change over time. Each of these women was interviewed 4 times over a 2-year period.

All analyses were conducted manually, in line with the interpretive nature of QLR (Neale, 2021). NT read all of the chosen case transcripts fully and coded inductively to generate open codes from the data. These codes were discussed in team meetings with DW, LM, TH, and MP, where interpretations were tested and refined collaboratively. Divergent views, such as whether sacrifice was economic or relational, were discussed until a shared understanding was reached. Table 1 illustrates the thematic framework.

**Table 1:** Thematic framework.

Sub-theme	Core experience	Case	Illustrative quote
Emotional rollercoaster	Non-linear emotional journey; joy, fatigue, stress, loneliness, and adaptation over time	Phumi	*"It is a rollercoaster of emotions. Sometimes I feel like I have everything figured out… then there are days I feel like I do not know what I am doing"*
Financial pressure	Dual burden of primary earner and caregiver; economic precarity; reliance on state grants	Mbali	*"Financially, emotionally, and physically, it's a very straining phase… there's no superwoman"*
Sacrifice and responsibility	Loss of personal goals, autonomy, and identity; deferred educational and career aspirations	Zinhle	*"Motherhood is just a part of your life you've practically given away… It's a happy sacrifice"*
Support	Presence or absence of family support as a critical buffer or compounding risk factor	Zama	*"When things are hard for me and I need to calm down, my mum is the one I turn to"*

### 2.7 Reflexivity

Reflexivity is essential in qualitative longitudinal research, as it acknowledges how the team's positionalities influence data collection and interpretation (Neale, 2021). The Trajectories team is highly interdisciplinary: NT, a Mozambican-born woman and an Anthropologist with experience in African health-seeking behaviour research, led the analysis with awareness of her outsider status; DW, a British psychologist, contributed to data collection and analysis while similarly reflecting on her outsider status. LM, and TH, familiar with the local communities, facilitated rapport-building and were integral to translation and maintaining meaning across languages; CG, a bioethicist and medical doctor, and NMK, a South African social worker and researcher, contributed with their disciplinary lenses to the analysis respectively; SAN and CED, both principal investigators in the Bukhali trial, provided valuable contextual input and qualitative analytical expertise; and MP, a medical anthropologist and senior researcher, contributed with the critical antrhopological perspective.

Interviews in multiple languages involved translation and reflection, with interpretation seen as a collaborative process shaped by social positions, theories, and responses, and acknowledging its role in generating knowledge. Across the interview waves, the interviewers developed familiarity and relationships with returning participants. This greatly facilitated rapport and trust between the participants and interviewers, resulting in more open conversations and richer data. The researchers did, however, need to reflect on how their familiar relationships could have influenced the data, for example, how their affective attitudes to participants could shape the analysis positively or negatively. Interviewers debriefed after each interview to reflect on these issues and maintain analytical consistency.

## 3 Participant demographics

QLR participants were aged 22–34 years at the time of Wave 1 data collection. All 60 participants are Black African women, of whom 51 were mothers of at least one child at the first interview. Of these, two women reported having three children, and one reported having four. In terms of education level, 43 participants had completed secondary school; 17 had not. Twenty-six participants had attained tertiary education, indicating varied educational trajectories within the cohort. Only 22 participants were employed. Of those engaged in work, three were self-employed, and one did voluntary work, highlighting limited access to stable formal employment. Most participants (46 women) relied on state social grants as their main source of income. This reliance on grants underscores the economic precarity experienced by many women in the cohort. Overall, the demographic profile illustrates a relatively young, economically constrained group of Black African women, with diverse educational backgrounds and high dependence on state social protection mechanisms. Table 2 illustrates the demographic characteristics of the study participants.

**Table 2:** Demographic characteristics of the QL research cohort (*N* = 60).

Characteristic	n (%)
Age at Wave 1	22–34 years
Ethnicity	
Black African	60 (100%)
Parity	
No children	9 (15.0%)
One child	48 (80.0%)
Three children	2 (3.3%)
Four children	1 (1.7%)
Education level	
Did not complete matric	17 (28.3%)
Completed matric	43 (71.7%)
Tertiary education^a^	26 (43.3%)
Employment status at Wave 1	
Formally/informally employed	22 (36.7%)
Self-employed	3 (5.0%)
Voluntary work	1 (1.7%)
Unemployed	34 (56.7%)
Primary source of income	
State social grants	46 (76.7%)
Employment income	22 (36.7%)

a Tertiary education is a subset of matric completers. Percentages may not total 100% due to overlapping categories. Source of income categories are not mutually exclusive.

## 4 Findings

Our data illustrate four key characteristics of young women's subjective experiences of motherhood in Soweto within the Bukhali cohort. First, the transition to motherhood is described as an ‘emotional rollercoaster’; second, as a time of financial pressure; third, as one that entails significant sacrifice; and fourth, as one that requires significant social support, especially for new mothers. Four emblematic cases are presented below to discuss each theme. Table 3 presents an overview of the four cases.

**Table 3:** Overview of the cases.

Characteristic	Zama	Mbali	Phumi	Zinhle
Children	two	three	one	one
Living situation	With the mother	With extended family	With the parents and sisters	With the mother and siblings
Employment	Volunteer informal work	Unemployed	Teacher's assistant	Self-employed
Income source	State grants informal	State grants (partial)	Salary	Self-employment
Partner support	Absent	Absent	Absent	Present but limited
Family support	Strong (mother)	Absent	Absent	Moderate (mother)
Education	Did not complete	Completed high school	Law degree completed	Uncompleted tertiary
Key theme	Emotional rollercoaster	Financial pressure	Sacrifice and responsibility	Support networks

### 4.1 Motherhood, a rollercoaster of emotions

Box 1 Case 1–Phumi.

**Table:** 

Phumi lives with her parents, three sisters, three of her older sister’s children, and a cousin. Her father is self-employed, running a shoe-repair business and serving as the family's primary financial provider; however, Phumi and her younger sister are also employed and contribute to the family's finances. Although she completed her law degree, she currently works as an educator's assistant in a primary school. Phumi aspires to be an attorney specialising in property law. During our first interview, Phumi revealed that she was five months pregnant, emphasising that she had never intended to become pregnant. “Before falling pregnant, I never wanted to have kids… it's a lot”. She characterised her motherhood journey as deeply emotional, noting that, apart from being unplanned, her pregnancy occurred at an inopportune moment, coinciding with her new job and relationship. Phumi believes that her pregnancy contributed to her partner's decision to end their relationship, as they had only been dating for two months before she became pregnant. She expressed regret and guilt over becoming pregnant so soon, stating that she could have taken measures to prevent it. This ultimately led to the dissolution of their relationship and the partner's disappearance. She recounted her pregnancy as sad, as she had to navigate the experience without the presence of the baby's father. In our second interview, with a two-month-old baby boy, Phumi expressed some happiness as she discussed the positive outcome of her pregnancy. She conveyed a mixture of emotions, acknowledging feeling overwhelmed by the necessity of caring for and being responsible for another life; she expressed fatigue, particularly due to the challenges of sleepless nights. However, she enjoyed the moments spent together. “I love breastfeeding, like, I think it is a beautiful thing, just sitting there holding my son. The eye contact, I think, is what I love.” Phumi described a mixture of good feelings and stressful moments. “It has been eight months now after having my son… It is a rollercoaster of emotions. Sometimes I feel like I have everything figured out, and it feels good. Then there are days I feel like I do not know what I am doing… Ja, that’s stressful”. She became emotional when discussing her unexpected status as a single mother and the absence of emotional support from the baby’s father. She voiced her frustration regarding the lack of anticipated support from her family, despite her sisters having experienced motherhood themselves. To manage the demands of work and motherhood, she decided to take the baby to daycare.

Participants articulated that motherhood is consistently accompanied by a spectrum of intense emotions, from immense joy and love for their children to overwhelming stress and anxiety. As described in Box 1, case 1, women characterised motherhood as a journey marked by fluctuations, with emotionally challenging moments that lead to fatigue and stress:

“It is hard. I don't want to lie, but it is hard…It is hard. Sometimes, it becomes too emotional. Sometimes it becomes too demanding. Sometimes it becomes stressful, like there is always something. Like, you can't just say, “Okay, today is just fine.” Okay, there are better days, but sometimes you feel like you can't do so much”. (Zama, Wave 2)

“It takes a lot to be me now as a mum. It needs someone who doesn't give up easily. You need to be strong because some of us don't take it easily. Some of us want to kill ourselves and run away from the truth, but you need to be strong” (Zama, wave 1).

This burden felt heavier for first-time mothers who hadn't initially expected to become mothers. Many participants experienced a “rollercoaster” of stress, leading to mixed feelings; for example, some who initially described motherhood as very demanding in the first interview had, by the third interview, embraced their role. They now saw motherhood as involving love, respect, understanding, and emotional availability for their children:

“Being a mom… yeah, it is loads of loving, understanding, accepting, open-minded, and respectful. So, it is everything”. (Zama, Wave 3).

The ‘emotional rollercoaster’ was exacerbated by the difficulties of discussing feelings; participants frequently described a household environment that discouraged emotional expression and hindered communication with their parents. They also described intergenerational disparities in approaches to emotional well-being, particularly maternal well-being.

“So, with my parents, I think it has to do with the different times we grew up in. They cannot understand you or what you are going through because times have changed, so it is very hard to connect with them emotionally…It's hard to communicate.” (Phumi, wave 2).

“Because she [mother] will say, ‘You are a mum, you have to suck it up and do whatever you need to do.’ So, our parents don't understand emotions. Like, when my daughter says, ‘Oh, I’m so tired, I think I’m depressed’, my mother would say, ‘What is depression? It is just being lazy’.” (Zama, wave 2).

The majority of the 60 participants we interviewed are single; some are in serious relationships that do not necessarily imply cohabitation, and very few women live with their partners. As a parent, they are affected by the absence of a partner in the child's life:

“you will be watching other girls going out to enjoy themselves while we are busy breastfeeding at home, with no baby daddy” (Zama wave 1).

Overall, our participants described their experiences of motherhood as emotionally turbulent and, at times, intensely ambivalent. It seems that moments of profound joy and fulfilment, such as bonding with their children, were held alongside challenging emotions, including loneliness and even despair. As such, participants highlight the complexity of maternal subjectivities, which cannot be reduced to a single emotion at any given moment.

### 4.2 Financial pressure

Box 2 Case 2: Mbali.

**Table:** 

Mbali grew up with her mother and three siblings. She lives with her three children (aged nine, five, and one year old), her mother, two sisters, one brother, two nephews, and four nieces (in total, eight children aged twelve, eleven, nine, eight, six, five, four, and a newborn baby boy). They live in informal housing without access to water and electricity. After finishing high school, she received a scholarship to attend a hair and beauty course. She was excited to attend the graduation ceremony, but she couldn’t as she had to help her sister with the funeral arrangements for her seven month old baby girl, who died from malnutrition. Mbali was unemployed during the first three interviews; she sometimes does hair calls at home to earn money. She doesn’t receive social grants for two of her children as their documentation is incomplete. She expressed the burden of providing food, affection, and attention to the kids. She explained that she ‘tolerates’ but doesn't like kids. She believes she is mothering all eight children in the house, as her sister is away most of the time. Her first child was born when she was 19 years old. She describes her pregnancy experience as ‘dramatic’: *“The [first] pregnancy wasn't nice, honestly. The guy started becoming abusive in a manner that I got arrested because of that. He started changing when I was six months pregnant, and then after I gave birth, I started noticing that he was into drugs.”* She started a new relationship and then fell pregnant. She was four months pregnant when she found out that he was married. His wife attacked her with a bottle. They broke up, and she never heard from him. Mbali found out about her third pregnancy during our second interview with our team, while we were discussing her health and well being, when she said, “I’m not sure how my health has been lately…actually, can you guys help me with a pregnancy test?” Mbali was able to access a pregnancy test through the Bukhali trial. Mbali describes the third pregnancy as unhealthy. She had iron problems, and the nurses advised that she or her baby could die in the ward if her iron levels didn't increase. She mentioned that she broke up with the married partner who impregnated her as he gave her money for an abortion, but she decided to keep the child for her conscience’s sake. *“I did not feel good killing a child”*. She was advised to eat well and take six iron tablets instead of 1 per day, but she couldn’t afford adequate meals: *“We would sleep on tea or porridge, no food some days”*. Her first two pregnancies were smooth deliveries, but the third pregnancy was a traumatic pre term delivery requiring an emergency Caesarean section. *“That pregnancy was stressful; I was not eating well…and the stress”*. After the delivery, her baby had complications and stayed in the intensive care unit for 5 days. The baby was very small and ill. From there, she was in and out of the hospital with the baby due to recurrent illnesses (infection, pneumonia, flu, breathing problems). By our third interview, Mbali was struggling with all the financial responsibilities (from food to education) for all the children in the household. She mentioned that she felt alone in the world because neither her mother nor her sisters were supportive. When she was admitted due to the newborn baby’s sickness, she had to leave the other children home alone and worried about them not having food, gas, wood to cook, and no one to take care of them. *“They survived alone”*, she said. Mbali expressed how she felt overwhelmed and exhausted as she couldn’t have a good sleep since the baby was born, which made her feel tired and anxious always, because she had no one to help. She described a turbulent relationship with her siblings and a pattern of unresolved household issues. She felt that her mother failed to distribute love and attention between her and her siblings, and this had become more pronounced as her mother is now old and mostly sick.

Most women in this QLR study were either unemployed or engaged in self-employment in the informal sector, such as bottle recycling, house calls for hair dressing and nails spa, selling clothes, food hampers, sweets, eggs, and cakes. Four of the 60 women held paid internships at large companies. Few participants worked in the formal sector (e.g., call centres, teacher assistants, retail), and their contracts were unstable, often lasting less than a year. This highlights labour market informality and instability as a core contributor to economic vulnerability among these women, a major source of mothers’ anxiety and stress. Mbali's case (Box 2) demonstrates the strong link between economic strain and subjective maternal well-being, particularly in a context of resource scarcity and her broader relational and care networks:

“I’m the mum of many, not just my children. It's a very hard phase. Financially, emotionally, and physically, it's a very straining phase.It sucks! It doesn't matter whether you're a superwoman at work; a superwoman wherever you go. When it comes to being a mother, there's no superwoman” (Mbali, wave 2)

Although most of the interviewed mothers receive the state child grant as designated caregivers, the grant is insufficient to cover the food, clothing, childcare, education and healthcare they want to provide for their children. In this sense, the data show that being a mother is not just a homemaking or nurturing role, as might be the case elsewhere, but rather a role that requires an entrepreneurial spirit to provide for one's children, often without a partner or family support. The burden of “having to provide no matter what” contributes to depression, anxiety and exhaustion. These women face dual burdens: they are both primary earners and primary caregivers.

The arrival of a child meant a focus on budgeting to constantly balance the cost of food, clothes, childcare, healthcare, education, and other expenses in precarious conditions:

“I am big on budgeting. My fear is not having enough money to provide for my son, so I would rather spend less and save. It's a huge financial pressure. Currently, I am still employed, so I think I save more than I spend. When I am not employed, I will save until I get my next job” (Phumi, wave 3).

Phumi elaborated on the fact that she must spend a lot of money and provide for her son, to the point that she makes every effort to be thrifty and to keep some provisions for the bad days:

“I'm not sure about my employment status in the next few months, as my contract is ending this month. I save for rainy days; that's the importance of saving (…). We need two packets of nappies a month, a big tin of formula, daycare expenses and his medicine. Because they grow so fast, we need a few clothing items every month” (Phumi, wave 3).

In these daily efforts to make ends meet, motherhood becomes inseparable from sacrifice and responsibility, as women consistently prioritise and worry about their children’s needs, often before their own.

### 4.3 Motherhood as sacrifice and responsibility

Box 3 Case 3: Zinhle.

**Table:** 

Zinhle grew up with her mother and siblings. At 22, she completed high school and went on to study psychology, but gave up university because she believed she was not ready. She is self employed, working with many businesses, including selling eggs and shakes at summer events and tutoring school aged children. She lives with her mother, four siblings, and their children. There are five children in the house: her daughter, her sister’s son, and two other children from her other sister. She has been in a three year relationship with the father of her eight month old baby girl. She characterised her pregnancy as positive. She became aware of the pregnancy when she enrolled in the Bukhali trial. She appreciated consulting the dietitian during pregnancy. Although the pregnancy was planned, it was not exactly what Zinhle had hoped for. When she found out she was pregnant, she felt overwhelmed and unprepared, while her partner was happy and ready. She went ahead with the pregnancy largely to please him, describing this as a difficult but necessary choice at the start of her journey into motherhood. Zinhle later spoke positively about her pregnancy and laughed as she recalled the birth of her child, which she described as the happiest moment of her life. At the same time, becoming a mother has changed her everyday life in quiet but meaningful ways. She no longer goes out as she used to, spends much less time with friends (she expressed the sacrifices she has made regarding her time with friends and outdoor activities), and feels especially tired at night when her baby struggles to sleep. Some of the changes include not going out anymore and spending less time with her friends. She also says that she is coping with her baby during the day, but not at night, as “She is not sleeping; she wants us to talk”, she says, laughing. She often shared these moments with humour, but they reveal the constant effort involved in caring for a young child. Although her partner supports her financially and emotionally and visits often, Zinhle carries most of the day to day responsibility for her baby. Looking ahead, Zinhle expressed her desire to return to university to study Anthropology and expand her businesses.

Most participants reflected on how they had to set aside personal ambitions, life goals and aspirations as they became mothers. Although they have mentioned the sacrifices (school, work, and other opportunities) they’ve made due to motherhood, most participants were still dreaming of the moment when they could achieve their goals, successes, and ambitions.

Zinhle's case (Box 3) illustrates the common sacrifices faced by some mothers in the study: a fractured sense of self, a loss of autonomy, and ambivalence about sacrificing personal aspirations. Some participants reflected on their limited time for self-care and its impact on their identities. One participant expressed this as a ‘happy sacrifice’:

“Motherhood for me is just a part of your life you’ve practically given away. It's no longer just you. You don't wake up in the moment and say, “I want to do this”. It's a happy sacrifice… I have to do it for my son… no longer only for you.” (Zinhle, wave 1).

Parenthood brought important changes for these young women regarding their independence and the opportunities they had lost. For example, Zinhle, when looking back, expresses that she does not regret becoming a mother but she is aware of what she has set aside for now:

“I don't regret being a mother. I love being a mother, but I wish I could've gone to varsity, done practicals, and stayed at res [university residence]. It hurts that I would have been living my own life without a baby”. (Zinhle, wave 1)

Phumi expressed similar sentiments around sacrifices and lost opportunities constrained by the burden of responsibility that came with motherhood.

“I just finished school and started my first job, so there are a lot of things I wanted to do before I could have a child, but now I can't.” (Phumi, wave 1).

“Yeah… the new me. Being responsible… I think there is a shift now because I have to be responsible for someone else. Even if I have to go somewhere, I know I have to come back home. Yeah, the responsibility is too much.” (Phumi, wave 2).

Most poignantly, women described how these lost opportunities were compounded by a lack of family or social support, or more positively, how it was support (especially from their own mothers) that helped them to cope.

### 4.4 Motherhood and support

Box 4 Case 4: Zama.

**Table:** 

Zama is a single mother of a twelve year old girl and a six year old boy. Neither father plays an active role in the children’s lives. Zama lives with her children and her mother, who helps raise the children and has a close relationship with her grandchildren. Zama works as a volunteer at her daughter’s school. In addition, she braids hair and babysits in her spare time to keep herself busy and earn money. She receives state grants for her kids and some support for her son from his father’s family. She did not complete her schooling and grew up an only child, raised by her mother after her father left when she was 7. Zama became pregnant with her first child at 17. While her boyfriend wanted an abortion, she chose to continue the pregnancy, as she felt that abortion was sinful. She was bullied by schoolmates, making the pregnancy emotionally difficult. Throughout this period, her mother was consistently present, supporting both her and the baby. The relationship with her child’s father later ended when she discovered his drug and alcohol use. Her second child was born when Zama was 21. She described this pregnancy and delivery as physically smooth, again emphasising her mother’s support. Although the father was involved during the pregnancy, the relationship ended when he was “not ready to be a father” and continued a lifestyle of “too many parties and nights out,” leaving Zama once more as the primary caregiver. Despite her mother’s ongoing financial and emotional support, Zama described single motherhood as emotionally demanding and often overwhelming. Zama’s account highlights how support from her mother makes motherhood possible yet does not remove the strain of raising two children largely on her own. In Zama’s story, motherhood is sustained through intergenerational support, but remains marked by emotional labour, instability in partner relationships, and the ongoing effort of coping day by day.

It was evident that most participants regarded a good support structure as crucial to overcoming the challenges they faced when becoming mothers. However, some of them experienced isolation and a lack of support as the example of Zama (Box 4, case 4).

For some participants, their mothers’ support was especially crucial:

“When things are hard for me, and I need to calm down, my mum is the one I turn to… My mum is there. She's very fond of children…She's the one who stays calm and relates very well with the children” (Zama, wave 1).

In contrast, other participants expressed a lack of family support and felt they were navigating motherhood alone:

“When I had just given birth, I felt least supported because I thought I would be held through motherhood, step by step: ‘This is what is expected of you, this is what you must do when this and that happens, this is how you should treat this’.. I had to navigate all that by myself. No one was there to support me. My mum, my older sisters, they have kids, they are mothers, they are home, but no one supported me.. I am just figuring it out on my own. I'm navigating motherhood alone (…). I use the internet. I also ask friends who have kids as well” (Phumi, wave 3).

“I can never count on my mum to make things easy. Yeah, I don't even know what is going to happen when I go to deliver. Since I count on no one being home to take care of the children, I don't even want to think about it. My mum will be there, but I know she will do nothing; those kids will be on their own until I go back. I count on no one… yeah” (Mbali, wave 3).

Overall, participants emphasised how social support, or the lack thereof, played a significant role in their experience of motherhood. It appears that the lack of support not only takes an emotional toll on mothers but also carries an extra cognitive burden for women who now need to ‘figure out’ and worry about childcare practices and dynamics. In contrast, as with the emblematic case of Zama, support alleviated her emotional and cognitive burden.

## 5 Discussion

Women described deep meaning alongside anxiety, exhaustion, and persistent worry. This emotional pattern aligns with evidence of high levels of psychological distress among mothers, particularly in low-resource settings. For example, data from South Africa show persistent symptom trajectories from pregnancy through five years postpartum (59). The nonlinear nature of emotional adjustment observed in this study echoes that reported by Shloim et al. (2020) and supports Kruger's (2020) argument that maternal identity develops gradually through experience. Our findings thus reinforce the importance of understanding matrescence as a critical life-course transition with direct implications for maternal mental health and reproductive wellbeing (31, 60–62).

The data also attest to how mothers often place their children's needs above their own, sacrificing personal ambitions, goals, and time, including stepping back from careers and working extra jobs, with consequences for their emotional well-being. In addition, given that our participants are mostly single mothers, many take on the responsibility of ensuring financial stability for their children and, in most cases, the entire extended family (63, 64). Support is provided, and how it is provided, as well as which networks exist and which are created, becomes critical not just for the mother, but also for female relatives.

The findings highlight the influential role of poverty-related risk factors, including unemployment, deprivation, disempowerment, and food insecurity, in shaping maternal mental health and subjectivities (22, 65). This consistent finding underscores the need to address these broader structural determinants alongside clinical interventions. The results also highlight the tension between sacrificing life goals and providing financially in a context of high unemployment, where most mothers rely on state social grants. These findings align with studies highlighting the role of structural inequalities in shaping maternal outcomes in South Africa (66, 67).

Finally, our findings show that trial participants felt supported by the trial, particularly in terms of health education and access to services (68). This is a noteworthy finding given that literature on South Africans’ attitudes to science and technology notes some degree of scepticism (69). However, none of the participants mentioned receiving support in navigating the transition to motherhood. This underscores the importance of designing interventions that not only support women but also address the specific challenges of matrescence as a distinct period in the life course.

## 6 Conclusion

To our knowledge, this is the first sustained, longitudinal engagement on maternal subjectivity in the context of Soweto. This study shows that motherhood in Soweto is experienced as a prolonged and demanding transition shaped by emotional strain, economic pressure, a sense of sacrifice, and uneven social support. Recognising lived experiences of motherhood is essential to improving maternal and reproductive health services. Programmes that focus solely on clinical outcomes risk overlooking key drivers of maternal wellbeing, caregiving capacity, and long-term child outcomes, especially in highly unequal contexts. Matrescence is a critical period in the life course, not only biologically, but also socially and psychologically, and we recommend that it ought to be recognised as a key life course event and process when designing early life interventions.

## Data Availability

The original contributions presented in the study are included in the article/Supplementary Material, further inquiries can be directed to the corresponding author.
